# 734. Abnormal Lipid Profiles in Human Babesiosis

**DOI:** 10.1093/ofid/ofab466.931

**Published:** 2021-12-04

**Authors:** Inderjit Mann, Sophia pham, Rachel Spector, Aikaterini Papamanoli, Evan Garry, Pooja Lamba, Sara Krivacsy, Michael D Lum, Aleena Zahra, Wei Hou, Eric Spitzer, Luis A Marcos

**Affiliations:** 1 Renaissance School of Medicine at Stony Brook University, Stonybrook, New York; 2 Renaissance School of Medicine, Merrick, New York; 3 Stony Brook University Hospital, Stony Brook, New York; 4 SUNY Stony Brook University Hospital, Stony Brook, New York

## Abstract

**Background:**

Babesiosis has gained attention as an emerging protozoal zoonotic disease with an expanding known incidence and geographical range in the US. The infection is caused by *Babesia microti* in the US and is transmitted by the bite of *Ixodes* ticks, and occasionally by blood transfusion. The diagnosis is usually established by microscopic examination of a stained blood smear to see intraerythrocytic organisms. The level of parasitemia is only loosely correlated with clinical severity. Anecdotal reports suggest that HDL cholesterol levels decline during acute babesiosis. In this study, we report cholesterol levels in a series of patients with acute babesiosis with the hypothesis that HDL levels may be a potential biomarker for more severe infections.

**Methods:**

A retrospective chart review was performed at Stony Brook University Hospital and Stony Brook Southampton Hospital between 2013 and 2018. Inclusion criteria was defined as a case of acute Babesia infection proven by peripheral blood smear microscopy and who had a lipid profile drawn during presentation to the emergency department. Cholesterol levels that were measured either before or after the infection (at least 1 month apart) were also recorded to compare to the levels reported during acute infection.

**Results:**

A total of 40 patients (27.5% female) met criteria for acute Babesia infection. Fifteen (37.5%) had a history of splenectomy. The patients were divided into two groups for comparisons based on the treating physician’s clinical decision: 32 patients who were admitted to the hospital and 8 patients who were not-admitted. History of hypertension was more common in admitted than non-admitted patients (37% vs. 17%, Chi-square test p=0.02); the median levels of LDL and HDL were more reduced in admitted than non-admitted patients (46 vs 76 mg/dL, p=0.04 and 9 vs 28.5 mg/dL, p=0.03, based on t-test respectively)

**Conclusion:**

LDL and HDL levels are significantly reduced in acute babesiosis, and LDL levels are inversely proportional to the parasitemia, suggesting that low levels of LDL may predict worsening disease in babesiosis. The mechanism of this phenomenon is unknown. Further prospective studies are needed.

**Disclosures:**

**All Authors**: No reported disclosures

